# Interaction of Macrophages and Cholesterol-Dependent Cytolysins: The Impact on Immune Response and Cellular Survival

**DOI:** 10.3390/toxins12090531

**Published:** 2020-08-19

**Authors:** Roshan Thapa, Sucharit Ray, Peter A. Keyel

**Affiliations:** Department of Biological Sciences, Texas Tech University, Lubbock, TX 79409, USA; roshan.thapa@ttu.edu (R.T.); sucharit.ray@ttu.edu (S.R.)

**Keywords:** *Streptococcus pyogenes*, *Clostridium perfringens*, streptolysin O, perfringolysin O, pore-forming toxin

## Abstract

Cholesterol-dependent cytolysins (CDCs) are key virulence factors involved in many lethal bacterial infections, including pneumonia, necrotizing soft tissue infections, bacterial meningitis, and miscarriage. Host responses to these diseases involve myeloid cells, especially macrophages. Macrophages use several systems to detect and respond to cholesterol-dependent cytolysins, including membrane repair, mitogen-activated protein (MAP) kinase signaling, phagocytosis, cytokine production, and activation of the adaptive immune system. However, CDCs also promote immune evasion by silencing and/or destroying myeloid cells. While there are many common themes between the various CDCs, each CDC also possesses specific features to optimally benefit the pathogen producing it. This review highlights host responses to CDC pathogenesis with a focus on macrophages. Due to their robust plasticity, macrophages play key roles in the outcome of bacterial infections. Understanding the unique features and differences within the common theme of CDCs bolsters new tools for research and therapy.

## 1. Introduction

Cholesterol-dependent cytolysins (CDCs) are a subset of pore-forming toxins that serve as key virulence factors for a wide range of lethal and opportunistic Gram-positive bacterial pathogens that collectively infect or invade nearly all parts of the human body. Consequently, hosts attempt to eliminate these pathogens with both general and tissue-specific approaches. One common approach that has tissue-specific flexibility is activation and polarization of macrophages. Macrophages coordinate the local tissue response with cytokines and can directly eliminate bacteria through phagocytosis and secretion of reactive oxygen/nitrogen species. They further promote wound repair and restore the tissue to homeostasis. As a result, pathogenic bacteria target macrophages for elimination, reprogramming, or shelter. CDCs figure prominently in many of these attempts. This review explores both general and specific molecular mechanisms used by CDCs to kill, control, or evade macrophages.

## 2. Cholesterol-Dependent Cytolysins

### 2.1. CDC-Producing Bacteria and Tissues They Invade

While CDCs are produced by a wide range of Gram-positive (and one Gram-negative) bacteria, the best-studied CDCs are produced by pathogenic *Streptococci*, *Clostridia*, and *Listeria* [1,2] (Table 1). *Streptococcus pyogenes* causes widespread pharyngitis but also causes potentially lethal infections such as the necrotizing soft tissue infection (NSTI) necrotizing fasciitis, streptococcal toxic shock syndrome, and septic cardiomyopathy [3,4]. Essentially all pathogenic clinical isolates of *S. pyogenes* produce the CDC streptolysin O (SLO), and deletion of SLO from *S. pyogenes* attenuates bacterial virulence in mice [5,6]. Similarly, *Streptococcus pneumoniae* is the leading cause of bacterial pneumonia and can cause otitis media and bacterial meningitis [7,8,9]. Most pathogenic isolates of *S. pneumoniae* produce the CDC pneumolysin (PLY), and deletion of PLY from *S. pneumoniae* attenuates bacterial virulence in mice [10]. *Streptococcus intermedius* is an opportunistic pathogen that produces the toxin intermedilysin (ILY) [11], while the pig pathogen *Streptococcus suis* produces the CDC suilysin (SLY) [12,13]. The opportunistic intracellular pathogen *Listeria monocytogenes*, which causes meningitis and miscarriage, is completely dependent on its CDC listeriolysin O (LLO) for virulence [14,15]. *Clostridium perfringens* causes the NSTI gas gangrene, which is exacerbated by its CDC perfringolysin O (PFO) [16,17]. *Clostridium tetani* is the causative agent of tetanus and secretes the CDC tetanolysin O (TLO), though the role of TLO in bacterial pathogenesis is unclear [18]. Similarly, *Bacillus anthracis* produces anthrolysin O (ALO), which shows hemolytic activity [19]. *Gardnerella vaginalis* is associated with bacterial vaginosis and produces the CDCs vaginolysin (VLY) [20,21,22]. Other pathogenic and nonpathogenic bacteria also produce CDCs, including inerolysin by *Lactobacillus iners* and lectinolysin (LLY) by *Streptococcus mitis*; however, their roles are not as well understood (reviewed in [2,23]). Thus, CDCs are common virulence factors expressed by human pathogens that cause many different diseases.

### 2.2. CDC Structure and Pore-Formation

**Figure 1:**
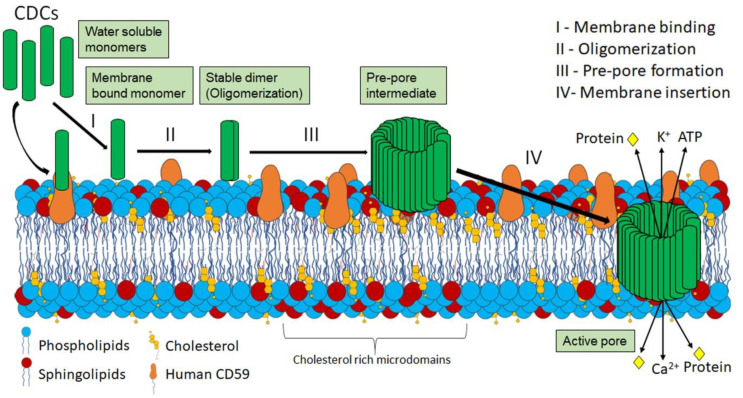
Pore formation by CDCs. (I) CDCs are secreted as water soluble monomers that bind the plasma membrane via their specific receptors (cholesterol for most CDCs, the GPI-anchored human CD59 for ILY, VLY, LLY). (II) Once bound, monomers initially dimerize to form stable dimer. (III) CDCs then start to oligomerize to form a pre-pore structure of ~35–50 monomers. (IV) After pre-pore formation, each monomer undergoes a coordinated conformational change to refold transmembrane helices into membrane-spanning β-strands. Collectively, these β-strands form a β-barrel in the membrane with a 25–30 nm diameter. Formation of these pores causes ion flux (Ca^2+^ influx and K^+^ efflux) as well as loss of cellular ATP and proteins.

CDCs are well-conserved at the protein sequence and structural levels across multiple organisms. They share a high degree of protein sequence and structural similarity (28–98%) [24]. Since CDC protein structure and pore-formation have been extensively reviewed elsewhere [1,2,24,25]), it is only briefly summarized here (Figure 1). CDCs contain four domains. Domains 1 and 2 are structural and retain contact with the aqueous environment during pore-formation [2,24,26,27]. Domain 3 includes the two transmembrane helices which convert to β-strands that penetrate the host membrane [2,24,26,27]. Domain 4 consists of the conserved Trp-rich undecapeptide and other structural loops involved in cholesterol sensing and membrane-binding [2,24,26,27]. CDCs are secreted as soluble monomers that orient on the host cell using human CD59 (ILY, VLY, and lectinolysin (LLY)) or possibly using glycans on the host cell, which facilitates the interaction of the L1-L3 loops in domain 4 with the sterol-rich host plasma membrane [27,28,29] (Figure 1). After successfully binding to the host cell membrane cholesterol, toxin monomers oligomerize into pre-pores composed of ~35–50 monomers [2]. Each monomer undergoes a coordinated conformational change, where the conserved undecapeptide in domain 4 interacts with the plasma membrane cholesterol [1,2,24,25]. This interaction drives refolding of the transmembrane helices into β-strands [1,2,24,25]. Insertion of the two β-strands into the membrane forms a β-barrel pore in the plasma membrane with a diameter of 25–30 nm [1,2,24,25]. The large pore then conducts both ions and proteins (Figure 1). While CDCs can lyse cells, in vivo, most CDCs are likely released at sublytic concentrations, enabling host cell survival [30]. However, CDC levels may vary in vivo because *S. pyogenes* strains overexpressing SLO kill neutrophils better than strains expressing normal amounts of SLO [31]. At sublytic doses, CDCs trigger several cellular processes including membrane repair, programmed cell death, the unfolded protein response, mitochondrial fragmentation, histone modification, and multiple innate and adaptive immune responses. Thus, even when CDCs fail to kill target cells, they elicit a wide variety of cellular changes.

### 2.3. Cellular Consequences of CDC Pore Formation

The best known outcome of CDC intoxication is cell lysis. Cell lysis is best characterized using erythrocytes. Erythrocytes are very sensitive to CDCs, easy to obtain, and release the easy-to-measure hemoglobin when lysed. Consequently, the capacity of the CDC to lyse a defined concentration of erythrocytes has been the measure of CDC activity for over one hundred years [32]. Erythrocytes are also far more sensitive to CDCs than nucleated cells because erythrocytes have an extremely limited ability to repair membrane disruptions [33,34]. Compared to erythrocytes, many nucleated mammalian cell lines, including HeLa, HEK, and 3T3 cells, are approximately 250–500 times more resistant to CDCs [35,36]. Interestingly, macrophages are even more resistant to CDCs than these cell types. Resting primary bone-marrow derived macrophages are ~10–20 times more resistant to SLO and PFO than HeLa, HEK, or 3T3 cell lines despite similar CDC binding [36,37]. Differentiation from monocytes to macrophages is associated with increased resistance to SLO [38]. Similarly, ALO kills primary macrophages poorly compared to THP.1 monocytes and neutrophils [39]. For PLY, the U937 macrophage cell line was more resistant than THP.1 monocytes and T cells [40,41], while neutrophils were more sensitive to PLY than macrophages [42]. Thus, macrophages are more resistant to CDCs than most other cell types.

**Figure 2:**
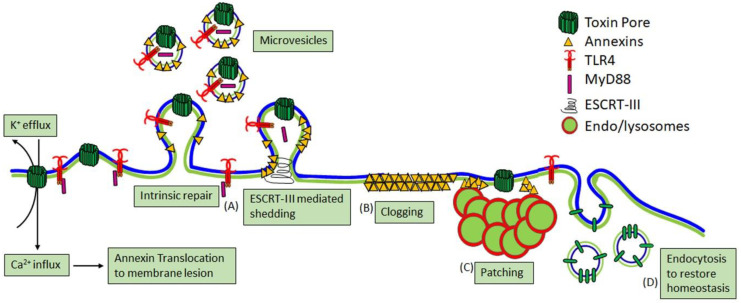
Major calcium-dependent membrane repair mechanisms. Nucleated mammalian cells utilize at least three (A–C) Ca^2+^-dependent membrane repair mechanisms. (**A**) Intrinsic repair is the spontaneous, lipid-dependent sequestration and shedding of toxin pores on microvesicles, which is enhanced by Endosomal Sorting Complex Required for Transport III (ESCRT-III)-mediated shedding. Shedding may also remove cellular proteins needed for signaling. (**B**) Clogging occurs when annexins form a crystalline lattice to seal off pore access to the cytosol. (**C**) Finally, patch repair is homo/heterotypic fusion of internal vesicles to form a membrane patch over the damaged membrane. (**D**) Endocytosis restores homeostasis after repair is completed by removing inactive toxins and unshed blebs.

It is unclear whether the increased resistance of macrophages to CDCs is due to reduced pore formation on the membrane relative to other cells or enhanced membrane repair processes. All nucleated mammalian cells have membrane repair responses that are primarily triggered by Ca^2+^ influx (reviewed in [43,44,45]). The three major repair mechanisms are patch repair, clogging, and intrinsic repair (Figure 2). While endocytosis was initially proposed as a repair mechanism [46], endocytosis now appears to act downstream of repair to help restore homeostasis by removing inactive toxins and sealed blebs that failed to shed [35,36] (Figure 2). Patch repair is the homo/heterotypic fusion of internal vesicles, especially endosomes and lysosomes with the plasma membrane to form a patch to reseal the damaged membrane [45]. Clogging is the recruitment of annexins and other proteins that create a lattice to block the pore [47,48,49,50,51]. Intrinsic repair is the lipid-dependent sequestration and shedding of toxin pores on microvesicles (MV) [35,36,52], which is enhanced by the Endosomal Sorting Complex Required for Transport III (ESCRT-III) machinery [53,54]. These repair mechanisms may act concurrently because annexins recruited to the damage site are shed along with toxin pores via microvesicles [49,52,55,56]. Thus, CDCs trigger several repair mechanisms that resist their lytic capacity.

Of these repair mechanisms, intrinsic repair is best described for CDCs because several of them, including SLO, PLY, ILY, and PFO, are shed on MV [35,36,52,54]. Shedding is triggered by toxin oligomerization [36]. Shed vesicles typically contain large quantities of toxin pores and prepores, along with glycosylphosphatidylinositol (GPI)-anchored proteins and repair proteins such as annexins and ESCRT [35,36,52,54,57]. The shedding rate appears to vary by toxin, with PFO triggering more robust shedding than SLO [55]. Interestingly, vesicle size may vary by cell type because vesicles shed from macrophages average smaller in size compared to those shed from other cells [36]. The size of shed vesicles may also inversely correlate with the number shed [41]. Toxin-laden vesicles have multiple downstream immune consequences (discussed in Section 3.4). Blebbing may preemptively confer resistance to CDCs. Resistance to SLO was associated with P2X7 receptor activation induced blebbing [58]. One caveat to this finding is that P2X7 itself increases membrane permeability to small dyes [59,60] and potentially activates small dye-conducting pannexin channels [61]. Overall, shedding is a conserved membrane repair response to resist CDC killing.

Along with lysis, CDCs may kill cells by programmed cell death mechanisms. Autophagy is induced by CDCs [62,63,64,65], though the outcome is CDC-dependent. In *C. elegans*, autophagy protects against SLO [62], while LLO helps *L. monocytogenes* evade autophagy [64]. Pyroptosis is a common cell death response in macrophages, driven by inflammasome activation (discussed in Section 3.2). LLO and PLY can drive apoptosis [66,67], while SLO and PLY can both kill by oncosis [68,69]. SLO, PLY, and LLO can all damage the mitochondria, leading to cell death [70,71,72]. SLO and PLY both reduce mitochondrial membrane potential, which leads to the oncotic death of the intoxicated cell [70,71], while LLO transiently fragments the mitochondrial network to interfere with cellular metabolism to promote *L. monocytogenes* infection [72]. Thus, CDCs can kill by direct lysis or by activating a programmed cell death pathway.

**Figure 3:**
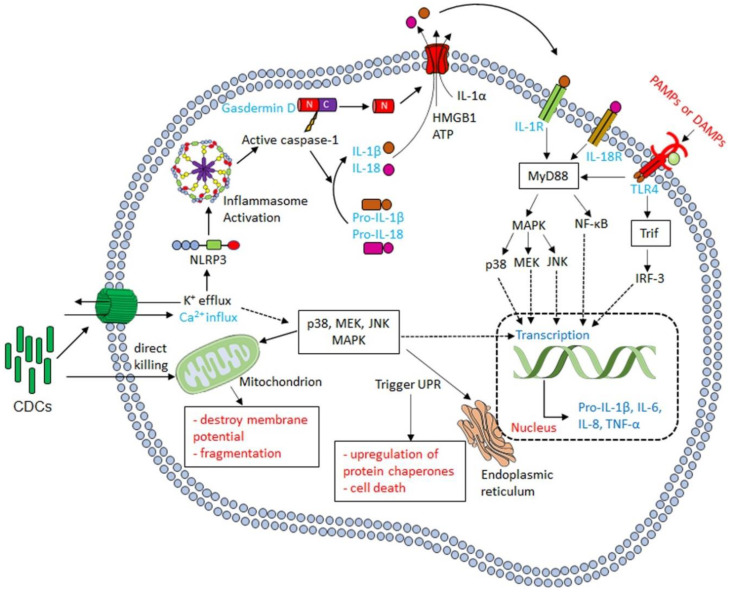
Cellular signaling after CDC intoxication. After successful pore formation on the plasma membrane, CDCs generate ionic flux. If CDCs do not lyse the cell outright, they can trigger multiple cellular mechanisms. Membrane damage activates the NLRP3 inflammasome, while ionic flux activates MAPK pathways, especially p38. Activation of p38 has many outcomes, including activation of the unfolded protein response and pro-inflammatory cytokine synthesis. Pro-inflammatory cytokines may also be made indirectly via danger-associated molecular patterns, including IL-1α, ATP, and high mobility group box 1 protein (HMGB1).

Programmed cell death is not the only signaling pathway activated by CDCs. CDCs also activate one or more Mitogen-activated Protein Kinase (MAPK) pathways (Figure 3). All three MAPK pathways can be activated by CDCs, but p38 activation is the most commonly reported. Activation of p38 following CDC intoxication occurs for SLO [73,74], PLY [73,75,76,77], LLO [78,79], ALO [73,80], VLY [22], and inerolysin [81]. Activation of p38 is likely a conserved cellular response to CDCs. While there is general agreement that sustained p38 activation occurs in the hours after intoxication, the upstream signaling pathways remain controversial. It is alternatively proposed that either Ca^2+^ influx [73] or K^+^ efflux [75,78,79] activate p38. However, the signaling intermediates between ion flux and p38 activation have not yet been determined. In contrast, the downstream signaling outcomes of p38 are better defined. Activation of p38 is considered protective because it promotes host survival [82], recovery of K^+^ after depletion by toxin [78,79], and pro-inflammatory cytokine/chemokine production [22,73,83]. The downstream effects for p38 are better described after PLY challenge. PLY-dependent cytoplasmic access of *S. pneumoniae* is limited by p38 [84]. Consequently, p38 is targeted by *S. pneumoniae*. PLY counteracts p38 by activating the deubiquitinating enzyme cylindromatosis (CYLD) [82]. CYLD inhibits p38, causing increased vascular leakage and acute lung injury [82]. Thus, p38 activation orchestrates long-term protection against CDCs.

Other MAPKs are also activated during CDC challenge. PLY can activate JNK [77,85], which blocks increased mucin production during *S. pneumoniae* infection [85]. Mucin production is protective, thus JNK activation benefits the pathogen [85]. However, JNK activation by PLY is antagonized by p38, maintaining protective mucin levels [85]. The MEK-ERK pathway is activated by LLO [78,79,86,87] and ALO [80]. For ALO, p38 and MEK contribute to syndecan shedding [80]. LLO activates MEK by Raf [86] and promotes cell survival [78], which facilitates invasion by the intracellular pathogen *L. monocytogenes* [87].

One outcome of MAPK activation by CDCs is activation of the unfolded protein response (UPR) in the endoplasmic reticulum (ER) (Figure 3). The UPR is a stress response that upregulates protein chaperones, regulates trafficking from the ER, and ultimately destroys the cell if the stress cannot be relieved [88]. While the UPR can destroy key cells in the body and promote cardiomyopathy following bacterial infection [89,90], activation of the UPR is generally deleterious to CDC-producing pathogens. The UPR protects against both *S. pyogenes* [91] and *L. monocytogenes* [92]. The UPR is triggered by LLO binding to the ER [93], despite the low fraction of cholesterol in the ER. While the ER is only ~5 mol% cholesterol, this cholesterol is accessible to CDCs [94]. Consequently, LLO can damage the ER and induce Ca^2+^ release from intracellular stores [95]. Ca^2+^ release promotes calpain activation and actin remodeling to promote repair [93]. However, too much intracellular Ca^2+^ is toxic, and during external PFT attack, the ER actively buffers elevated intracellular Ca^2+^ levels [47]. Thus, intracellular signaling via the ER protects cells from CDC-induced death.

In addition to activating the UPR, CDCs induce several post-translational modifications. CDCs stimulate ubiquitination, SUMOylation, and histone modification in a cell-type specific fashion [96,97,98]. LLO targets the ubiquitin pathway in HeLa cells but does not target this pathway in the Raw264.7 macrophage cell line [96]. Targeting ubiquitin and ubiquitin-like proteins may be conserved across CDCs because LLO, PFO, PLY, and SLY all degrade the E2 SUMO ligase Ubc9 after K^+^ efflux [97,98]. Impairment of Ubc9 is associated with improved infection [97]. Interestingly, the changes in SUMOylated proteins varied by CDC, suggesting CDCs fine-tune this process [97]. CDCs also trigger histone modifications. PFO, PLY, and LLO dephosphorylate Ser 10 in histone H3 and deacetylated histone H4 [99]. K^+^ efflux, but surprisingly not pore formation, is needed to induce these changes [99,100]. Overall, CDCs induce a variety of cellular changes due to pore formation.

Finally, CDCs modify the host vasculature, which is critical for the spread of bacterial infection and the infiltration of immune cells such as macrophages. ALO disrupts gap junctions [80,101], while PLY contributes to vascular infiltration of *S. pneumoniae* [102]. PFO may impact blood pressure and vasoconstriction, though it was not replicated [103,104]. CDCs further impair immune recruitment. SLO can promote platelet aggregation and microvascular occlusion, which blocks myeloid cell entry [71,105], while both SLO and PLY block neutrophil migration [106,107,108]. PFO also interferes with neutrophil recruitment to the site of gas gangrene [109]. A second pore-forming toxin in *S. pyogenes*, Streptolysin S, stimulates pain neurons, which release the neuropeptide calcitonin gene-related peptide to block neutrophil migration [110]. Overall, CDCs help bacteria disseminate while interfering with recruitment of myeloid cells.

## 3. CDC Interactions with Macrophages

While many of the previously described mechanisms are common to most nucleated cells, including macrophages, there are also several macrophage and myeloid-specific responses to CDCs. Macrophages detect the CDCs or their effects as signs of infection and attempt to respond to the pathogen. However, the pathogens evade many of these responses, either using their CDC directly or using other closely expressed virulence factors. In macrophages, CDCs generally stimulate cytokine production and promote inflammasome activation. However, CDCs also contribute to immune evasion by interfering with phagocytosis and hijacking membrane repair to blunt inflammatory responses. Finally, macrophages and other antigen-presenting cells present peptides from CDCs to stimulate protective adaptive immunity. While some of these responses are well-described for many CDCs, the data supporting other responses by macrophages have only been described for a few CDCs. We predict that future studies will bear out the generality of these responses, in contrast to specific activities of individual CDCs described in Section 4.

### 3.1. Cytokine Production in Response to CDCs

One key consequence of activating the signaling pathways discussed in Section 2.3, especially the p38 pathway, is production of pro-inflammatory cytokines. The major pro-inflammatory cytokines induced by CDC challenge are Tumor Necrosis Factor α (TNFα,) Interleukin (IL)-1β, IL-6, and IL-8, though other cytokines and chemokines are also CDC-dependent (Figure 3). However, one challenge with CDC-dependent cytokine production is ascertaining if the cytokine is produced by signaling pathways directly stimulated by the CDC or indirectly by other danger- or pathogen-associated molecular patterns (DAMPs and PAMPs). Many studies attributing cytokine production to CDCs used bacteria with and without deletion of the CDC. Since CDCs are often key to bacterial virulence, it is difficult to discern if changes in pro-inflammatory cytokine production are due to overall reduced virulence or directly due to the toxin. Pure CDCs may contain contaminants, such as toll-like receptor (TLR) ligands, which complicate interpretation of data. Often studies do not include inactive toxins to control for the presence of any contaminants in purified toxins. Cellular damage by CDCs further releases several DAMPs, including IL-1α, ATP, and high mobility group box 1 protein (HMGB1) [111,112,113,114], which may exert autocrine and paracrine effects on cells, including TLR engagement and pro-inflammatory cytokine production (Figure 3). These effects may be especially apparent when longer (12+ h) time points are used. Finally, signaling pathways can be activated independently of plasma membrane receptors by the Ca^2+^ fluctuations that occur during membrane damage and repair [47]. Thus, extreme care should be taken in interpreting pro-inflammatory cytokine production in response to CDCs.

Direct cytokine production in response to CDCs is triggered by inflammasome (see Section 3.2), p38, and Ca^2+^ dependent pathways. Activation of p38 by SLO, PLY, ALO, and VLY leads to IL-8 production [22,73,77]. Activation of p38 also induces secretion of macrophage migration inhibitory factor (MIF) by PLY, which helps reduce the bacterial load [83]. Finally, p38 also stimulates TNFα production after SLO challenge [115]. TNFα is critical for recruiting macrophages during a subcutaneous *S. pyogenes* infection, which limits bacterial dissemination [116]. CDCs also stimulate IL-6 production. LLO, PLY, and SLY stimulate IL-6, which is Ca^2+^ dependent for LLO and PLY [117,118,119,120]. Similarly, LLO and PLY trigger IL-1α release and calpain activation in a Ca^2+^ dependent fashion [113,114]. Overall, CDCs can activate pro-inflammatory cytokine signaling.

The ability of CDCs to directly stimulate TLR signaling pathways remains controversial. While some studies suggest that PLY [121,122], ALO, LLO, SLO, and PFO [123] all trigger TLR4, other studies have not observed TLR4-dependent responses [124,125]. Similarly, NF-ΚB activation, which is downstream of TLRs, is variably reported for CDCs. Some studies observe NF-ΚB activation by CDCs [126,127], while another did not [125]. One potential explanation for the discrepancy is the autocrine and the paracrine effects involving TLRs and/or IL-1 receptor (IL-1R), which also signals through MyD88. LLO-induced NF-ΚB is IL-1R dependent [128]. PLY-induced TNFα production and TLR4 activation were measured at 24 h [129,130], thus TLR4 activation could occur secondary to DAMP release. There are also cell type differences in cytokine production in response to CDCs. For example, PLY induces opposite effects for TNFα production in dendritic cells and macrophages [131]. Overall, TLR activation may be secondary to other effects of CDCs.

In some cases, it is clear that CDCs indirectly lead to cytokine and chemokine production. PLY induces CCL2 and CCL5 indirectly [132]. PLY-dependent interferon (IFN) β, IL-23, and granulocyte-macrophage colony-stimulating factor (GM-CSF) production depends on other pneumococcal factors [133]. In particular, PLY permits entry of pneumococcal DNA, which triggers a host-protective IFNβ response [134,135]. Finally, in other cases, it remains unclear if cytokine or chemokine production is direct or indirect. For example, SLO stimulates cytokine-induced neutrophil chemoattractants and macrophage inflammatory protein (MIP)-1α [136]. PLY similarly induces MIP-1α, which helps recruit neutrophils to the lung [119]. Both LLO and PLY trigger NO production, which is dependent on the IFNγ receptor for PLY [120,137]. Dissecting the differences in cell-type and toxin-specific cytokine responses remains an active area of research.

### 3.2. CDCs Activate the Inflammasome

**Figure 4:**
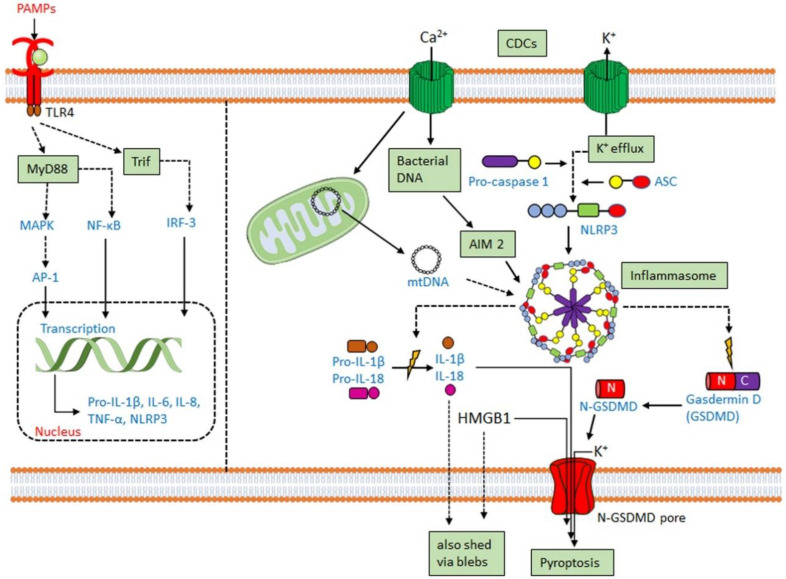
Inflammasome activation by CDCs. Macrophages and other myeloid cells primed by toll-like-receptor (TLR) ligation upregulate pro-IL-1β and NLRP3. When primed macrophages are perforated by CDCs, K^+^ efflux activates the NLRP3 inflammasome. CDCs may also activate theAIM2 inflammasome either via import of bacterial DNA or destabilization of the mitochondria to release mitochondrial DNA (mtDNA). When activated, NLRP3 or AIM2 oligomerize and recruit ASC. ASC recruits pro-caspase-1, which autoactivates. Once active, caspase-1 cleaves pro-IL-1β, pro-IL-18, and gasdermin D (GSDMD). The N-terminus of GSDMD forms pores in the membrane, enabling cytokine release and pyroptosis.

Along with the previously discussed cytokines, CDCs promote IL-1 secretion [130,138,139]. IL-1β is usually secreted following activation of the inflammasome. The inflammasome is a multiprotein complex comprising a cytoplasmic sensory pattern-recognition receptor, the scaffolding protein apoptosis-associated speck-like protein containing a CARD (ASC)/Pycard and an inflammatory caspase (caspase-1 or caspase-11 in mouse, caspases-1,4 or 5 in human) that promotes the inflammatory cell death process pyroptosis and the activation/release of pro-inflammatory cytokines IL-1β and IL-18 (reviewed in [111,140]) (Figure 4). Sensory pattern-recognition receptors that activate the inflammasome include proteins in both the Pyhin-family and the nod-like receptors (NLRs). They sense various pathogen-associated molecular patterns and danger-associated molecular patterns, including membrane perforation by CDCs [111,140]. Membrane perforation is sensed by nucleotide-binding oligomerization domain-like receptor family, pyrin domain-containing 3 (NLRP3), presumably indirectly via loss of K^+^ [141], while cytoplasmic bacterial or mitochondrial DNA is sensed by absent in melanoma 2 (AIM2) [142] (Figure 4). After activation, NLRP3 or AIM2 oligomerize ASC, which recruits and activates caspase-1. Activated caspase-1 then cleaves the pore-forming toxin gasdermin D to promote pyroptosis (Figure 4). Pyroptosis prevents intracellular pathogens from sheltering in the cell and releases pro-inflammatory mediators, including HMGB1, IL-1β, and IL-18 [111,140]. The inflammasome promotes anti-pathogen responses after sensing the CDC challenge.

CDCs activate two inflammasomes. CDCs directly activate the NLRP3 inflammasome via membrane perforation and K^+^ efflux, and some also indirectly activate AIM2 by facilitating the entry of mitochondrial or bacterial DNA into the cytosol. PFO [143], SLO [37,144], TLO [125], PLY [102,145], LLO [146,147,148], and SLY [149] all activate the NLRP3 inflammasome. LLO-mediated phagosomal rupture and lysosomal permeabilization further activate NLRP3 [147]. Bacteria deficient in LLO [148,150], PLY [102,145], PFO [143], and SLO [144] fail to stimulate IL-1β production. Other toxins, including streptolysin S [69] and *C. perfringens* α-toxin [143], fail to activate the inflammasome. Thus, CDCs are necessary for pathogen sensing by the NLRP3 inflammasome.

AIM2 activation has functional redundancy with NLRP3 in responding to CDCs. AIM2 can be activated by either bacterial DNA or mitochondrial DNA following *L. monocytogenes* infection or PLY intoxication [70,84,151,152,153]. AIM2 is also activated by mitochondrial DNA released into the cytosol after cholesterol perturbations [154]. Upregulation of the enzyme cholesterol-25-hydroxylase (Ch25h) by type I or type II interferons (IFNs) produces the regulatory oxysterol, 25-hydroxy-cholesterol [155]. The 25-hydroxy-cholesterol regulates cholesterol biosynthesis, flux, and storage [156,157]. Deletion of Ch25h from macrophages leads to increased IL-1β production in response to LPS-mediated type I interferon (IFN) induction [158]. When IFNs are unable to induce Ch25h, macrophages undergo cholesterol overload and switch to aerobic glycolysis with mitochondrial damage [154]. Mitochondrial damage permits the escape of mitochondrial DNA into the cytosol, activating the AIM2 inflammasome to overproduce IL-1β [154]. Consequently, Ch25h deletion confers AIM2-dependent protection from *L. monocytogenes* infection on macrophages [154,159].

Interestingly, macrophage release of 25-hydroxy-cholesterol confers the opposite phenotype upon epithelial cells because macrophage-derived 25-hydroxy-cholesterol protects them from *L. monocytogenes* spread [159]. In epithelial cells, 25-hydroxy-cholesterol reduces the amount of CDC-accessible cholesterol in the plasma membrane by activating acyl CoA: cholesterol acyltransferase (ACAT) [159]. ACAT triggers the internalization and esterification of CDC-accessible cholesterol, which reduces the fraction of cholesterol available to pathogens and CDCs without modifying total cholesterol levels [159]. This reduction in accessible cholesterol prevents the intercellular spread of *L. monocytogenes* [159], presumably due to reduced ability of LLO to damage the plasma membrane. Indeed, PFO, ALO, and SLO all show reduced binding and membrane damage in IFN-stimulated macrophages, which is due to 25-hydroxy-cholesterol reducing the fraction of CDC-accessible cholesterol [160]. In a skin model, 25-hydroxy-cholesterol reduced the extent of damage from CDCs [160]. Thus, immune cells use oxysterols to modify their plasma membrane cholesterol to protect their plasma membrane and the plasma membrane and the barrier function of non-immune cells as well as to limit inflammasome activation.

Inflammasome activation is generally beneficial to the host. While extracellular pathogens might benefit from pyroptotic macrophage death due to removal of effector cells, the pro-inflammatory cytokine secretion, especially the neutrophil-recruiting IL-1β, is detrimental to extracellular pathogens [161,162,163]. However, one exception may be *C. perfringens* myonecrosis, where PFO-dependent pathology requires inflammasome activation [143]. NLRP3 activation and ASC expression are protective during *S. pneumoniae* infection [102,145,151]. IL-1β is protective in lethal pneumococcal mouse infections [164], while elimination of IL-18 reduces inflammation and improves survival time in a lethal mouse meningitis model [165]. IL-1 is protective during *S. suis* [149] and *S. pyogenes* [163] infections. Consistent with *L. monocytogenes*’s susceptibility to IFNγ and Th1 responses, it is more sensitive to IL-18 than to IL-1β [166]. Consequently, inflammasome activation is one target of pathogen immune evasion. Often, the pathogens use a related virulence factor to target either inflammasome activation, IL-1β, or IL-18. When these pathogens are insensitive to loss of IL-1β or IL-18, it is likely that other virulence factors promote evasion of inflammasome activation.

### 3.3. CDCs Damage Phagosomes and Permit Phagolysosomal Escape

Phagocytosis is the cellular engulfment process of large particles (>0.5 µm in diameter). It is used by innate immune cells such as macrophages, dendritic cells, and neutrophils to internalize and kill extracellular pathogens [167]. Intracellular pathogens typically hijack phagocytosis to prevent phagosome fusion with the lysosome and may escape into the cytosol. Indeed, *L. monocytogenes* requires LLO for phagosomal escape and for preventing fusion with lysosomes [168,169,170]. While it is known that transgenic expression of other CDCs such as PFO can promote the escape of *L. monocytogenes* [171] or *Bacillus subtilis* [172,173], it is now appreciated that other traditionally extracellular bacteria such as *S. pyogenes* and *C. perfringens* rely on CDC-dependent phagosome interference to promote infection [174,175,176,177]. However, phagosomal escape is not perfect [178], thus it is not an all-or-none process. Interestingly, CDCs variably stimulate or impair phagocytosis. LLO stimulates ion flux, which promotes the internalization of *L. monocytogenes* [179,180]. Conversely, SLO interferes with phagocytosis [181]. However, after membrane damage and repair, compensatory endocytosis is activated to restore homeostasis [36,182]. Before phagosomal escape, LLO, PFO, and PLY may also interfere with acidification in non-macrophages [169]. However, lysosomal membrane permeability stimulates inflammasome activation in macrophages [183,184,185]. Consequently, when PLY interferes with lysosomal acidification in macrophages, it also drives cell death [186]. Interestingly, these events appear to occur independently of phagosome escape. Importantly, in macrophages, LLO permeabilizes phagosomes to small molecules prior to large ones, which led the authors to conclude that LLO forms pores of different sizes, possibly due to insertion of incomplete pores [170]. However, ESCRT-III mediates phagosomal membrane repair [187], thus an alternative explanation is that repair mechanisms limit the extent of damage caused by LLO, preventing the loss of larger molecules. While the structure of LLO is optimized for activity at low pH [188], the host protein gamma-interferon inducible lysosomal thiolreductase (GILT) further activates LLO by reducing the single cysteine in the protein [189]. Thus, CDCs extensively target phagocytosis to evade cell death and promote escape and immune evasion.

### 3.4. CDC-Mediated Innate Immune Evasion

While CDCs activate several cell defense mechanisms, they also contribute to evading immune activation. Immune evasion is best described for SLO and PLY. CDCs interfere with phagocytosis and inhibit cytokine production. SLO reduces phagocytosis and *S. pyogenes* killing by neutrophils [181]. Similarly, PLY-stimulated pyroptosis of neutrophils lead to elastase release, which blocks phagocytosis of *S. pneumoniae* in the inflammasome-defective macrophage cell line Raw264.7 [42]. SLO may stimulate the ubiquitination and the degradation of IL-1β [190]. Similarly, PLY expression reduces maturation of human DCs and pro-IL-1β, IL-8, and IL-12p70 production in response to *S. pneumoniae* [191]. Finally, PLY, PFO, and SLO can block TNFα production [124,131]. Thus, CDCs promote immune evasion.

At least three mechanisms have been described for CDC-mediated immune evasion. CDCs may target reactive oxygen formation and the respiratory burst. Both LLO and PFO block NADPH oxidase localization to the phagosome [192]. Similarly, SLO blocks the respiratory burst in neutrophils [193]. This blockade of reactive oxygen species also interfered with elastase secretion, IL-8 production and neutrophil extracellular trap formation [193]. A second mechanism of cytokine inhibition is engagement of the CDC by the Mannose receptor (CD206). Infection of primary human monocyte-derived DCs with PLY-deficient *S. pneumoniae* increased TNFα production [131]. In contrast, infection of THP.1-derived macrophages or primary neutrophils did not show this phenotype [131]. PLY-mediated cytokine inhibition was attributed to PLY engagement of CD206, which triggered SOCS1 [131]. CD206 binding to PLY did not require glycans [131]. Finally, after membrane repair, SLO and PFO transiently blocked TNFα production and macrophage upregulation of the activation marker CD69 and costimulatory protein CD86 in response to TLR4 or IFNγ [124]. Shedding of immune receptors such as IFNγR1 and TLR4 and signaling adapters such as MyD88 on microvesicles partly accounted for the phenotype [124]. However, intrinsic repair was not sufficient to block TNFα production because pore formation was needed to inhibit TNFα [124]. It remains to be determined if interference with cholesterol-rich microdomains accounts for the remaining inhibition. Thus, there are multiple mechanisms by which CDCs interfere with immune function.

Finally, the vesicles shed during membrane repair may also have immunomodulatory capacity. The toxin-laden microvesicles are readily phagocytosed by macrophages [194]. If phagocytosis of microvesicles occurs in the presence of IFN or IFN-inducing TLRs, macrophages accumulate neutral lipids, forming a foam cell phenotype in a peroxisome proliferator-activated receptor α (PPARα) dependent manner [195]. This is consistent with the ability of IFNs to enhance cholesterol esterification [196]. The involvement of Ch25h in microvesicle-triggered foam cell formation was not investigated. Importantly, microvesicles shed from toxin-challenged cells reduced TNFα production and activation of T cell lines [195]. HEK-derived microvesicles or artificial liposomes laden with PLY polarized macrophages into a CD14^+^MHCII^low^CD86^low^ phenotype while increasing IL-6, TNFα and IL-1β [197]. After challenge with these vesicles, macrophages were more responsive to TLR2 ligands but showed reduced TLR4 activation [197]. In contrast to PLY, SLO-laden vesicles stimulated CD14^+^MHCII^hi^CD86^hi^ macrophages that produced low levels of IL-6 and were more responsive to TLR4 [197]. While the mechanistic difference between these vesicles laden with SLO and PLY has not been established, the CD14^+^MHCII^low^CD86^low^ phenotype in PLY-vesicle stimulated macrophages suggests SOCS1 upregulation [198]. One possibility is that the difference in phenotypes is due to a PLY-CD206 interaction that upregulates SOCS1. Indeed, in a mouse model, *S. pneumoniae* colonization of the upper respiratory tract induced the CD206^+^, wound healing alternatively-activated M2a macrophages that reduced influenza disease severity [199]. Overall, CDCs act to suppress cytokine production both in target cells and in responding macrophages.

### 3.5. CDCs as an Adaptive Immune Target

While CDCs try to impair macrophages, CDCs are popular targets for antigen presentation by macrophages and other APCs to the adaptive immune system. Bacteriocidal T and B cell responses to LLO are mounted against *L. monocytogenes* [200,201], LLO-expressing *Escherichia coli* [202], or *B. subtilis* [203]. Memory CD4^+^ T cells respond to PLY [204] and are associated with an absence of *S. pneumoniae* carriage [205]. Antibodies to ALO [206], PFO [207], or SLY [12] protect mice from lethal infections. Antibodies to CDCs are readily produced [118,200,206,208,209,210]. Anti-SLO and anti-PLY titers can be detected in human serum [210,211], indicating CDCs are robustly antigenic in humans. Importantly, anti-CDC T and B cell responses do not depend on hemolytic activity [209,212], which suggests that non-hemolytic CDC toxoids may serve as useful vaccine targets. A PLY toxoid has been used for vaccination against *S. pneumoniae*. The anti-PLY toxoid vaccine was well tolerated in a Phase I trial [213]. However, no additional benefit from including the PLY toxoid was observed in Gambian infants when it was included with pneumococcal histidine triad protein D (PhtD) in the vaccine [214]. Similarly, an immunogenic and well-tolerated PLY/PhtD vaccine showed no improvement over the current capsular vaccine in preventing otitis media in Native American infants [215]. Overall, CDCs are targeted by both innate and adaptive immune systems.

## 4. Individual CDCs

These broad responses by macrophages to CDCs are further modified by each toxin. Where macrophage-specific information is lacking, we discuss findings in related cells that may inform their interaction with specific CDCs.

### 4.1. Streptolysin O (SLO)

Since SLO is one of the archetypal CDCs, many of its activities have already been described above. SLO drives the virulence of pandemic *S. pyogenes* and is upregulated in pandemic strains [216,217]. Elimination of SLO from *S. pyogenes* reduces mortality in a mouse model of invasive disease [5]. SLO is sufficient to trigger cardiac arrhythmia by uncoupling the electrical pacing of cardiomyocytes [4]. Due to its importance as a virulence factor, SLO has been targeted with erythrocyte-derived “nanosponges” to improve macrophage and neutrophil survival [218]. Thus, SLO is a key virulence factor.

SLO differs from other CDCs in the rate at which it binds to the membrane. SLO binds to lipid membranes faster than PFO [219,220]. This binding is governed by the lipid-binding L3 loop because the binding rate can be changed to that of PFO by point mutations in the lipid-binding L3 loop [219,220]. Interestingly, changing the mode of membrane binding does not alter the rate or extent of vesicle shedding during membrane repair [55]. This suggests that differences in oligomerization or pore insertion may drive shedding responses, which is consistent with oligomerization, but not binding, triggering intrinsic repair [36]. Overall, a comparative analysis of CDC binding, oligomerization, and pore formation will continue to deliver new insights into CDC function.

Perhaps the most unique feature of SLO is its extended N-terminus. This N-terminus promotes the cytosol-mediated translocation of the cytotoxic virulence factor *S. pyogenes* NAD glycohydrolase (NADase) [221,222]. Cytosol-mediated translocation is the ability of SLO to transfer of NADase into the cytosol [221,222]. Cytosol-mediated translocation requires the N-terminus of SLO, but does not require pore formation [223]. Even beyond physical interaction [224], SLO and NADase work closely together. Both toxins share the same operon in *S. pyogenes* [225] and synergize to increase cytotoxicity [221,222]. NADase enhances SLO binding to the membrane independently of SLO domain 4 cholesterol or glycan interactions [226]. Both toxins impair lysosomal acidification of phagosomes containing *S. pyogenes* and promote intracellular survival [175,227]. NADase also cleaves IL-1β to neutralize that cytokine [228]. The SLO N-terminus helps SLO and NADase synergize to promote *S. pyogenes* virulence.

Apart from its role in cooperating with NADase, SLO shares some functional redundancy with the other hemolytic toxin produced by *S. pyogenes*, Streptolysin S. Streptolysin S and SLO are both cytotoxic to macrophages and neutrophils [71,181]. Knocking out both SLO and streptolysin S shows milder disease phenotypes beyond knocking out either toxin alone [71,181,229]. Streptolysin S cooperates with SLO during early stages of infection to induce necrotic lesions and remove resident tissue macrophages in mice [181,229]. Streptolysin S is better at targeting neutrophils during infection [110,230,231]. Streptolysin S blocks neutrophil recruitment via neuron-released calcitonin gene-related peptide [110]. Thus, SLO synergizes with other key virulence factors and acts as a potent virulence factor during *S. pyogenes* infection.

### 4.2. Pneumolysin (PLY)

PLY contributes to many of the diseases caused by *S. pneumoniae*. While *S. pneumoniae* can be carried asymptomatically, it is a leading cause of lethal bacterial pneumonia, otitis media, meningitis, and cardiomyopathy [7,8,9]. The presence of PLY can drive the switch between chronic and lethal lung infections in mice [232]. Indeed, PLY cytotoxicity is sufficient to drive the lethal effects of pneumonia [30]. In conjunction with pneumococcal surface protein A (PspA), PLY is necessary for hearing loss during otitis media [233]. PLY contributes to severe meningitis by triggering glutamate release from astrocytes, which causes glutamate-dependent toxicity [234]. The PLY-dependent activation of PKCα-troponin and UPR pathways reduces the contractile properties of cardiomyocytes, causing acute cardiac injury [89]. While PLY is necessary for cardiomyopathy and macrophage-dependent necroptosis, PLY is not sufficient because these phenotypes depend on the *S. pneumoniae* strain [235]. PLY is one potential therapeutic target because liposomes engineered to deplete PLY were protective in a mouse infection model [236]. However, one limitation to mouse models of pneumococci is that murine LDL, but not human LDL, inactivates PLY [211]. This could lead to underestimating the virulence of PLY, or overestimating the success of anti-PLY therapies in murine models of *S. pneumoniae* infection. Overall, PLY is critical for many diseases caused by *S. pneumoniae*.

*S. pneumoniae* infections are fought in part by macrophages. Macrophages secrete TNFα, which is needed to stop *S. pneumoniae* infections [237]. PLY stimulates macrophage nitric oxide generation, which promotes antimicrobial killing [238]. This shows the importance of these PLY-stimulated responses to bacterial control and why the pathogen goes extensive efforts to evade these responses. In addition to cytokine production discussed in Section 3, PLY stimulates other host responses. PLY causes Ca^2+^-dependent increases in prostaglandin E_2_ and leukotriene B_4_ by human neutrophils in vitro, interfering with inflammatory responses during pneumococcal infection [239]. PLY can activate classical and alternate pathways of complement independently of pore formation [240,241]. PLY can bind to the Fc portion of antibodies [242]. These features help *S. pneumoniae* deplete host complement and increase bacterial dissemination [243,244]. PLY stimulates cellular phospholipase A and A2 and elastase release in neutrophils in a Ca^2+^-independent fashion [245,246]. Thus, PLY has wide-ranging impacts on myeloid cells designed to neutralize these important cells.

### 4.3. Perfringolysin O (PFO)

PFO, also known as *θ*-toxin, promotes the virulence of *Clostridium perfringens*, especially during the NSTI gas gangrene. PFO interferes with the recruitment of leukocytes during gas gangrene. While high doses of PFO are leukocidal, lower doses impair the chemotactic migration and morphology of neutrophils [109]. PFO further induces vascular leukostasis, blocking immune cell recruitment to the site of infection during gas gangrene [16,109,207,247]. Finally, sublytic concentrations of PFO disrupts neutrophil cytoskeletal polymerization and disassembly while upregulating adherence proteins [109]. PFO exacerbates gas gangrene by limiting the response of myeloid cells.

Insight into the mechanisms by which PFO limits myeloid cells has come from learning how PFO engages the host plasma membrane. PFO is strictly dependent on cholesterol for binding [248]. Consequently, many cholesterol sensors are non-hemolytic variants of PFO or only its cholesterol-binding domain 4 [1]. However, PFO can engage only about one-third of the total plasma membrane cholesterol [249]. This PFO-binding pool of cholesterol is defined to be “accessible” cholesterol [249]. Accessible cholesterol is cholesterol not complexed with sphingomyelin or other proteins and lipids [249]. Cholesterol accessibility provides a powerful framework for understanding cholesterol distribution in the plasma membrane. However, the relationship between cholesterol accessibility, liquid-ordered membrane domains, and cholesterol-rich microdomains remains unclear. For example, the pre-pore of PFO has a higher affinity for cholesterol-rich microdomains than the transmembrane pore [250]. The definition of accessible cholesterol may further vary depending on the CDC used. For example, the binding and pore formation of PFO are distinct from SLO. PFO binds more slowly than SLO, and insertion of the β-barrel takes longer [219]. This slower binding and insertion may be due to more restrictive lipid microenvironment needs for PFO over SLO [219]. Alternatively, differences in putative glycan requirements could drive differences in binding and insertion [29]. Consequently, PFO takes longer than SLO to achieve full toxicity in cells [55]. Thus, while PFO has taught us about CDC engagement of cholesterol in the membrane, care needs to be taken in interpreting cholesterol-binding studies using PFO or its domain 4 variants.

The interactions of PFO with the membrane in vivo are even more complex. During gas gangrene, PFO acts synergistically with another *C. perfringens* virulence factor, α-toxin [16,247]. α-toxin is a zinc metallophospholipase that cleaves phosphatidylcholine and sphingomyelin [251]. Cleavage of sphingomyelin by α-toxin releases cholesterol from sphingomyelin-cholesterol complexes and enhances PFO toxicity [251]. The activity of α-toxin presumably helps *C. perfringens* neutralize the protective accessible-cholesterol-reducing effect of Ch25h, but this remains to be tested. PFO and α-toxin together subvert the host immune response by limiting the extravasation of inflammatory cells into the site of the infection [16,176,247,252]. PFO limits extravasation in several ways. In a mouse myonecrosis model, PFO promotes aggregation of leukocytes and platelets in the microvasculature, which blocks circulation and immune cell access [176]. Both α-toxin and PFO upregulate adhesion molecules on the surface of inflammatory cells, which enhances intravascular cell aggregation and promotes vascular occlusion [253,254]. Neutrophil-dependent vascular occlusion could account for the deleterious effects to the host of inflammasome activation in a mouse model of *C. perfringens* induced myonecrosis [143]. In contrast, inflammasome activation is typically host protective against other CDC-producing pathogens. While myonecrosis is largely dependent on α-toxin, the contribution of PFO was NLRP3 dependent [143]. Inflammasome activation in neutrophils triggered by PFO could enhance or initiate aggregation of leukocytes and platelets, causing occlusion and limiting immune cell access. However, this hypothesis remains to be tested. Overall, PFO cooperates with α-toxin to modify the host membrane to cause pathogenesis during gas gangrene.

### 4.4. Listeriolysin O (LLO)

Unlike the other pathogens described here, *Listeria monocytogenes* is an intracellular pathogen that requires its CDC, LLO, for intracellular survival. LLO helps *L. monocytogenes* escape from the macrophage phagolysosome [168,170]. *L. monocytogenes* mutants lacking LLO fail to escape phagolysosomal killing and do not grow intracellularly [168]. Intracellular growth and intercellular spread enable *L. monocytogenes* to cause miscarriage/premature birth or meningitis in susceptible populations [14,15]. In the placenta and the fetus, *L. monocytogenes* triggers a Th1 response, which induces labor and either miscarriage or premature birth [255]. In contrast, the mechanism by which *L. monocytogenes* causes meningitis is not fully understood. LLO is considered a neurotoxin [256], but the extent to which its neurotoxicity drives meningitis is not known. Thus, LLO is key to *L. monocytogenes* pathogenesis and intracellular survival.

Due to the primarily intracellular lifestyle of *L. monocytogenes*, LLO contains key differences from other CDCs. Most notably, LLO has a strong pH dependence with maximal activity around pH 5.5 [257]. A triad of acidic residues in domain 3 confers the pH sensitivity and denatures LLO at >30 °C and pH 7 [188]. However, cholesterol stabilizes LLO at neutral pH [258]. This stabilization enables LLO to damage the plasma membrane. Plasma membrane damage by LLO secreted from intracellular bacteria is limited by the presence of the PEST sequence in LLO [259]. The PEST sequence promotes clathrin-mediated endocytosis of LLO [259]. However, localized damage from LLO promotes intercellular spread [260]. Intercellular spread is limited by the 25-hydroxycholesterol-mediated reduction in accessible plasma membrane cholesterol [159]. It is not clear if access to membrane cholesterol is increased by *L. monocytogenes* phosphatidylinositol-specific phospholipase C (PlcA) or broad-range phospholipase C (PlcB). Overall, LLO promotes *L. monocytogenes* infection and spread by targeting phagosomal and plasma membranes.

When targeting host membranes, LLO cooperates with PlcA and PlcB. PlcA, PlcB, and LLO are all regulated by the master transcription factor PrfA [261]. LLO and the phospholipases cooperate to promote phosphatidylinositol metabolism in endothelial cells [262], phagosomal escape [263], and evasion of autophagy [64,264,265]. The phospholipases increase the extent and the number of calcium spikes in the J774 macrophage cell line that occur during bacterial invasion [266]. However, phosphocholine produced by PlcB can inhibit LLO activity, providing a negative feedback loop for LLO [267]. Overall, PlcA and PlcB contribute to remodeling the membrane to facilitate the precise amount of LLO-induced damage needed for *L. monocytogenes* spread.

One potential key difference between LLO and other CDCs may be a distinct membrane repair response. LLO might trigger different membrane repair responses due to its primary role in phagosomal escape and intercellular transfer. The evidence suggesting a distinct repair response is based on a reduced impact of calcium on cellular survival. While small pore-forming toxins such as aerolysin did not show calcium dependence [78], CDCs such as SLO, PFO, or PLY generally triggered calcium-dependent repair mechanisms [35,47,55,56,268]. In contrast, reduction or lack of calcium did not increase cell death from LLO to the same extent that it increased PLY-dependent killing [269,270]. While reducing calcium from 2 mM to ~1 mM or 0 mM increased PLY killing of glial cells up to 10-fold, it improved LLO-mediated killing only 2.5-fold [269,270]. This was attributed to a decrease in the extent of vesicle shedding triggered by LLO compared to PLY, though shedding was observed for both CDCs [270]. However, these findings could be cell-type specific because HeLa cells did not show an increase cell death between 2 mM or 0.4 mM calcium when challenged with PFO or SLO [55]. SLO and PFO also have different shedding rates, yet both are calcium sensitive [55]. The cell type specific differences might be due to different cystic fibrosis transmembrane conductance regulator (CFTR) levels or activity because CFTR facilitates LLO activity [271]. Overall, this suggests that a comparative analysis of CDCs would help us better understand CDC-specific functions.

### 4.5. Anthrolysin O (ALO), Tetanolysin O (TLO) and Suilysin (SLY)

Both ALO and TLO make relatively minor contributions to the virulence of their respective pathogens. ALO, produced by *Bacillus anthracis*, contributes to the pathogenesis of *B. anthracis* and intracellular survival in immune cells [101,272]. ALO lyses monocytes, macrophage, and neutrophils [39]. ALO works together with three phospholipase C proteins produced by *B. anthracis* and can compensate for the deletion of these lipases in a murine model of anthrax [272]. ALO may promote gut epithelial disruption, much like PFO [101]. As with PFO, the domain 4 of ALO has been used to detect cholesterol on the plasma membrane of cells, including macrophages [160,273,274] However, the domain 4 of ALO may be more stable and better tolerate maleimide-based fluorophore addition than the PFO domain 4 [94,160,273], so it is emerging as the preferred CDC-derived cholesterol sensor over PFO. TLO, produced by *Clostridium tetani*, is presumed to contribute to *C. tetani* pathogenesis by destroying host cells. TLO can cause cardiac failure in mice [275]. TLO is lytic against bone marrow derived macrophages (BMDM) and platelets [194,276]. TLO seems to bind membranes similarly to PFO in the absence of sphingomyelin [249,277]. Since these CDCs are not central to the virulence of their respective pathogens in humans, less is known about their impact on the immune system. It remains to be determined if their role in targeting immune cells, especially macrophages, is similar to other CDCs or fulfills unique tissue-specific roles for these pathogens.

While TLO and ALO are dispensable for pathogenesis, SLY is essential for the pathogenesis of *Streptococcus suis* in mice [278]. However, in pigs, SLY may be dispensable for invasive disease [118,278]. Similar to PLY, SLY helps *S. suis* escape complement mediated killing and phagocytosis by reducing opsonization [279,280]. However, opsonization of *S. suis* increases bacterial adherence to macrophages and SLY-dependent cytotoxicity [281]. SLY remodels the host cell cytoskeleton by activating RhoA and Rac1 GTPases [282]. Moreover, as with PFO, SLY induces platelet-neutrophil complexes in a Ca^2+^ dependent manner [283]. Improving our understanding of the impact of SLY on pig macrophages during *S. suis* pathogenesis is important for understanding its contribution to disease in pigs and may help us learn which aspects of CDC functionality underwent diversification in Streptococci compared to CDCs in other species.

### 4.6. Intermedilysin (ILY), and Vaginolysin (VLY)

In contrast to most CDCs, one subset of CDCs, including ILY, VLY, and lectinolysin (LLY), produced by *Streptococcus intermedius*, *Gardnerella vaginalis*, and *S. mitis*/*S. pseudopneumoniae*, respectively, requires human CD59 for membrane binding [11,22,28,284,285]. While ILY requires human CD59, VLY can bind cholesterol even without CD59, though at a lower affinity [286]. ILY, VLY, and LLY still require cholesterol for pore formation [28] and represent one toolset to dissect the role of cholesterol in binding and pore formation in CDCs. Due to this unique binding nature, these CDCs have been used as an alternative to diphtheria toxin depletion in mice and explored as anti-cancer agents in humans. Both ILY and LLY have been engineered to deplete cells [287,288]. Monocytes, macrophages, T cells, and dendritic cells in the spleens of *Lck-Cre^+^ x ihCD59^+^* and *Cd11c-Cre^+^ x ihCD59^+^* mice are targeted by ILY [288]. The ILY domain 4 has been fused to other proteins to target them to CD59^+^ cancer cells [289]. Similarly, LLY binds to glycans that are upregulated on cancer cells, such as the Lewis y antigen [287]. Thus, improving our understanding of how these CDCs interact with macrophages and other immune cell types will help us better exploit them for therapy.

Interestingly, the CD59-binding CDCs are produced by bacteria that regularly reside in the oral or the vaginal cavities without causing disease. However, *S. intermedius* can cause brain and liver abscesses [290], and *S. mitis* can cause endocarditis, septicemia, and other complications [291]. *G. vaginalis* is more directly associated with bacterial vaginosis [292]. Most clinical isolates of *G. vaginalis* from bacterial vaginosis patients express VLY [20]. VLY promotes IL-1β release from epithelial cells, and more IL-1β is stimulated from bacteria that reach the basolateral surface [293]. The increased cytokine production is due to the increased amount of CD59 expressed on the basolateral surface [293]. Interestingly, VLY also stimulates microvesicle shedding from the apical surface [294]. Presumably, VLY is present on these shed blebs. Shedding from the apical surface may preserve epithelial integrity and reduce inflammation. How macrophages respond in a VLY-dependent fashion to *G. vaginalis* remains to be determined.

## 5. Conclusions

Our understanding of CDC function has come a long way from simple cell lysis to appreciating the complex effects they have on both immune and non-immune cells. Understanding the unique features and differences of individual toxins within the common framework of CDC biology has already generated new tools for research, therapeutics to treat multiple bacterial infections, and mechanistic pathways of resistance to pore-forming toxins. While many host responses to CDCs, including calcium influx, potassium efflux, p38 activation, and membrane repair, are likely conserved across cell types, immune cells also directly sense CDCs to fight their causative organism. Elimination of CDC-producing pathogens relies on myeloid cells, including macrophages. While many myeloid and macrophage-specific responses have been elucidated, especially inflammasome activation, cytokine production, and phagocytosis, other mechanisms remain to be discovered and explored. The interactions between the multiple pathways known and their tissue- and/or CDC-specific impacts infection are exciting areas for future work. Similarly, comparative analyses of CDCs will continue to reveal key insights into CDC function and how they target macrophages. The mechanism of macrophage resistance to CDCs also remains an open question. Identifying these pathways and their interactions will develop new approaches to dealing with CDC-producing pathogens.

## Figures and Tables

**Table 1:** Summary of cholesterol-dependent cytolysins (CDCs) discussed and subset of diseases.

Toxin Name	Abbreviation	Organism	Diseases
Streptolysin O	SLO	*Streptococcus pyogenes*	Necrotizing fasciitis, septic shock, cardiomyopathy, pharyngitis
Pneumolysin	PLY	*S. pneumoniae*	Pneumonia, meningitis, otitis media
Perfringolysin O	PFO	*Clostridium perfringens*	Gas gangrene
Listeriolysin O	LLO	*Listeria monocytogenes*	Meningitis/Miscarriage
Suilysin	SLY	*S. suis*	Meningitis/septicemia
Anthrolysin O	ALO	*Bacillus anthracis*	Anthrax
Tetanolysin O	TLO	*C. tetani*	Tetanus
Intermedilysin	ILY	*S. intermedius*	Brain/liver abscess
Vaginolysin	VLY	*Gardnerella vaginalis*	Bacterial vaginosis
Lectinolysin	LLY	*S. mitis and S. pseudopneumoniae*	Endocarditis/septicemia
